# 4-(Imidazol-1-yl)benzoic acid

**DOI:** 10.1107/S1600536811003345

**Published:** 2011-01-29

**Authors:** Zheng Zheng, Wen-Qian Geng, Zhi-Chao Wu, Hong-Ping Zhou

**Affiliations:** a Department of Chemistry, Anhui University, Hefei 230039, People’s Republic of China, and Key Laboratory of Functional Inorganic Materials Chemistry, Hefei 230039, People’s Republic of China

## Abstract

In the title mol­ecule, C_10_H_8_N_2_O_2_, the imidazole and benzene rings form a dihedral angle of 14.5 (1)°. In the crystal, inter­molecular O—H⋯N hydrogen bonds link the mol­ecules into chains extending in [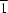
01], which are further linked into sheets parallel to (102) through weak C—H⋯O inter­actions.

## Related literature

The crystal structures of the Cd and Co complexes with the title mol­ecule were described by Gao *et al.* (2009 ▶) and Zhang *et al.* (2007 ▶), respectively.
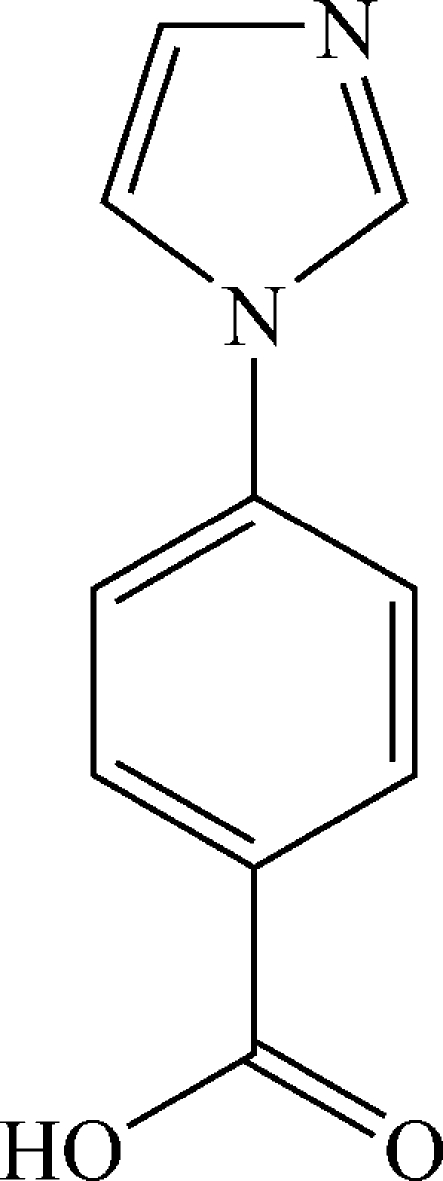


## Experimental

### 

#### Crystal data

C_10_H_8_N_2_O_2_
*M* *_r_* = 188.18
Monoclinic, 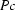

*a* = 4.1443 (11) Å
*b* = 6.6561 (19) Å
*c* = 15.706 (4) Å
β = 101.023 (7)°
*V* = 425.3 (2) Å^3^
*Z* = 2
Mo *K*α radiation
μ = 0.11 mm^−1^
*T* = 296 K
0.30 × 0.20 × 0.20 mm

#### Data collection

Bruker SMART CCD diffractometer
Absorption correction: multi-scan (*SADABS*; Sheldrick, 1996 ▶) *T*_min_ = 0.969, *T*_max_ = 0.979
2483 measured reflections
782 independent reflections
626 reflections with *I* > 2σ(*I*)
*R*_int_ = 0.032

#### Refinement

*R*[*F* ^2^ > 2σ(*F* ^2^)] = 0.040
*wR*(*F* ^2^) = 0.104
*S* = 0.71
782 reflections
128 parameters
2 restraints
H-atom parameters constrained
Δρ_max_ = 0.14 e Å^−3^
Δρ_min_ = −0.17 e Å^−3^

### 

Data collection: *SMART* (Bruker, 2002 ▶); cell refinement: *SAINT* (Bruker, 2002 ▶); data reduction: *SAINT*; program(s) used to solve structure: *SHELXTL* (Sheldrick, 2008 ▶); program(s) used to refine structure: *SHELXTL*; molecular graphics: *SHELXTL*; software used to prepare material for publication: *SHELXTL*.

## Supplementary Material

Crystal structure: contains datablocks I, global. DOI: 10.1107/S1600536811003345/cv5031sup1.cif

Structure factors: contains datablocks I. DOI: 10.1107/S1600536811003345/cv5031Isup2.hkl

Additional supplementary materials:  crystallographic information; 3D view; checkCIF report

## Figures and Tables

**Table 1:** Hydrogen-bond geometry (Å, °)

*D*—H⋯*A*	*D*—H	H⋯*A*	*D*⋯*A*	*D*—H⋯*A*
O1—H1⋯N2^i^	0.82	1.83	2.645 (5)	178
C9—H9⋯O2^ii^	0.93	2.42	3.332 (6)	168

Symmetry codes: (i) 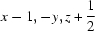; (ii) 
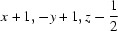
.

**Fig. 1.**
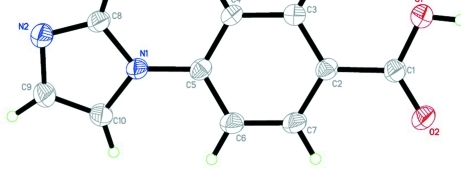
The molecular structure of (I) showing 30% probability displacement ellipsoids.

## supplementary crystallographic information

### Comment

The molecules of the title compound, (I), are often used as coordinating ligands in the metal complexes (Gao *et al.*, 2009; Zhang *et al.*, 2007). Herewith we presenet the crystal structure of (I).

In (I) (Fig.1), the imidazole ring is twisted out of the plane of benzene ring at 14.5 (1)°. In the crystal structure, intermolecular O—H···N hydrogen bonds (Table 1) link the molecules into chains extended in direction [-101]. These chains are further linked into sheets parallel to the plane (102) through the weak C—H···O interactions (Table 1).

### Experimental

A 150 ml round-bottom flask was charged with a magnetic stirrer and a reflux condenser, iminazole (44 mmol), K_2_CO_3_ (6.00 g, 43 mmol), 30 ml DMSO and a little Aliquat 336 were added. 4-fluorobenzaldehyde (4.5 ml, 42 mmol) was added dropwise to the mixture at 363 K and stirred for 15 min. Then the reaction mixture was refluxed for 24 h at 353 K, cooled to room temperature, poured into 150 ml ice-water and filtered. The primrose yellow crude product was obtained, washed with distilled water, and dried *in vacuo* at room temperature, then purified by recrystallization with ethyl acetate to give the desired analytical pure intermediate products. Intermediate product (12.5 mmol) and 15 ml 20% (wt) NaOH (aq) were added to a round-bottom flask equipped with a magnetic stirrer and a reflux condenser at 333 K for 30 min. Then AgNO_3_ (4.00 g, 24 mmol) was added to the mixture group by group. The reaction mixture was refluxed for 24 h at 333 K, cooled to room temperature and filtered. Excessive HCl (1 *M*) was added to the filtrate and adjust pH to 2, a great deal of sediments were obtained and then filtered. The crude product was recrystallized with ethanol. 4-imidazolylbenzoic acid: Yellow crystals. Weight: 1.44 g, Yield: 64%.

### Refinement

All hydrogen atoms were placed in geometrically idealized positions (O—H = 0.82 Å, C—H = 0.93 Å) and constrained to ride on their parent atoms, with *U*_iso_(H) = 1.2 *U*_eq_(C) or 1.5 *U*_eq_(O). Due to the absence of any significant anomalous scatterers in the molecule, the 408 Friedel pairs were merged before the final refinement.

### Crystal data

**Table:**

C_10_H_8_N_2_O_2_	*F*(000) = 196
*M**_r_* = 188.18	*D*_x_ = 1.470 Mg m^−^^3^
Monoclinic, *P**c*	Mo *K*α radiation, λ = 0.71073 Å
*a* = 4.1443 (11) Å	Cell parameters from 553 reflections
*b* = 6.6561 (19) Å	θ = 2.6–21.0°
*c* = 15.706 (4) Å	µ = 0.11 mm^−^^1^
β = 101.023 (7)°	*T* = 296 K
*V* = 425.3 (2) Å^3^	Prism, yellow
*Z* = 2	0.30 × 0.20 × 0.20 mm

### Data collection

**Table:**

Bruker SMART CCD diffractometer	782 independent reflections
Radiation source: fine-focus sealed tube	626 reflections with *I* > 2σ(*I*)
graphite	*R*_int_ = 0.032
φ and ω scans	θ_max_ = 25.5°, θ_min_ = 2.6°
Absorption correction: multi-scan (*SADABS*; Sheldrick, 1996)	*h* = −3→4
*T*_min_ = 0.969, *T*_max_ = 0.979	*k* = −7→8
2483 measured reflections	*l* = −18→19

### Refinement

**Table:**

Refinement on *F*^2^	Primary atom site location: structure-invariant direct methods
Least-squares matrix: full	Secondary atom site location: difference Fourier map
*R*[*F*^2^ > 2σ(*F*^2^)] = 0.040	Hydrogen site location: inferred from neighbouring sites
*wR*(*F*^2^) = 0.104	H-atom parameters constrained
*S* = 0.71	*w* = 1/[σ^2^(*F*_o_^2^) + (0.0601*P*)^2^ + 0.4878*P*] where *P* = (*F*_o_^2^ + 2*F*_c_^2^)/3
782 reflections	(Δ/σ)_max_ < 0.001
128 parameters	Δρ_max_ = 0.14 e Å^−^^3^
2 restraints	Δρ_min_ = −0.17 e Å^−^^3^

### Special details

**Table:**

Geometry. All e.s.d.'s (except the e.s.d. in the dihedral angle between two l.s. planes) are estimated using the full covariance matrix. The cell e.s.d.'s are taken into account individually in the estimation of e.s.d.'s in distances, angles and torsion angles; correlations between e.s.d.'s in cell parameters are only used when they are defined by crystal symmetry. An approximate (isotropic) treatment of cell e.s.d.'s is used for estimating e.s.d.'s involving l.s. planes.
Refinement. Refinement of *F*^2^ against ALL reflections. The weighted *R*-factor *wR* and goodness of fit *S* are based on *F*^2^, conventional *R*-factors *R* are based on *F*, with *F* set to zero for negative *F*^2^. The threshold expression of *F*^2^ > σ(*F*^2^) is used only for calculating *R*-factors(gt) *etc*. and is not relevant to the choice of reflections for refinement. *R*-factors based on *F*^2^ are statistically about twice as large as those based on *F*, and *R*- factors based on ALL data will be even larger.

### Fractional atomic coordinates and isotropic or equivalent isotropic displacement parameters (Å^2^)

**Table:**

	*x*	*y*	*z*	*U*_iso_*/*U*_eq_	
O1	0.4877 (9)	−0.2462 (5)	0.4370 (2)	0.0588 (9)	
H1	0.4273	−0.2969	0.4789	0.088*	
O2	0.5637 (9)	0.0207 (5)	0.52362 (19)	0.0599 (10)	
N1	1.0752 (8)	0.3543 (5)	0.1902 (2)	0.0415 (9)	
C9	1.2725 (12)	0.5824 (7)	0.1140 (3)	0.0569 (13)	
H9	1.3408	0.7049	0.0952	0.068*	
C2	0.7016 (11)	0.0470 (6)	0.3847 (3)	0.0413 (10)	
C4	0.8260 (11)	0.0591 (6)	0.2410 (3)	0.0444 (11)	
H4	0.8267	−0.0025	0.1878	0.053*	
C7	0.8116 (11)	0.2437 (7)	0.3973 (3)	0.0499 (11)	
H7	0.8012	0.3079	0.4493	0.060*	
C3	0.7080 (10)	−0.0435 (7)	0.3054 (3)	0.0462 (11)	
H3	0.6322	−0.1745	0.2954	0.055*	
N2	1.2859 (9)	0.4028 (6)	0.0725 (2)	0.0518 (10)	
C6	0.9360 (11)	0.3462 (7)	0.3342 (3)	0.0491 (12)	
H6	1.0147	0.4766	0.3443	0.059*	
C1	0.5766 (11)	−0.0580 (6)	0.4551 (3)	0.0465 (11)	
C5	0.9428 (9)	0.2532 (6)	0.2556 (3)	0.0399 (10)	
C8	1.1662 (11)	0.2706 (7)	0.1202 (2)	0.0466 (11)	
H8	1.1463	0.1343	0.1071	0.056*	
C10	1.1460 (13)	0.5559 (7)	0.1859 (3)	0.0548 (12)	
H10	1.1128	0.6547	0.2252	0.066*	

### Atomic displacement parameters (Å^2^)

**Table:**

	*U*^11^	*U*^22^	*U*^33^	*U*^12^	*U*^13^	*U*^23^
O1	0.088 (3)	0.047 (2)	0.0476 (17)	−0.0050 (18)	0.0288 (17)	−0.0015 (14)
O2	0.094 (3)	0.049 (2)	0.0417 (17)	0.0028 (19)	0.0252 (18)	−0.0047 (16)
N1	0.050 (2)	0.0351 (18)	0.041 (2)	0.0025 (17)	0.0151 (16)	−0.0019 (17)
C9	0.074 (4)	0.043 (3)	0.059 (3)	−0.003 (2)	0.025 (2)	0.006 (2)
C2	0.045 (2)	0.042 (3)	0.037 (2)	0.006 (2)	0.0100 (17)	−0.0013 (19)
C4	0.055 (3)	0.045 (3)	0.036 (2)	−0.001 (2)	0.0162 (19)	−0.0062 (19)
C7	0.068 (3)	0.041 (3)	0.042 (2)	0.004 (2)	0.014 (2)	−0.008 (2)
C3	0.056 (3)	0.040 (2)	0.045 (2)	−0.003 (2)	0.014 (2)	−0.0038 (19)
N2	0.063 (3)	0.048 (2)	0.047 (2)	−0.0019 (19)	0.0190 (17)	0.0003 (18)
C6	0.065 (3)	0.038 (2)	0.045 (3)	0.001 (2)	0.013 (2)	−0.006 (2)
C1	0.059 (3)	0.039 (3)	0.042 (2)	0.006 (2)	0.010 (2)	0.003 (2)
C5	0.042 (2)	0.040 (2)	0.038 (2)	0.0084 (18)	0.0089 (18)	0.0003 (19)
C8	0.059 (3)	0.041 (2)	0.042 (3)	0.002 (2)	0.015 (2)	−0.0038 (18)
C10	0.072 (3)	0.036 (2)	0.064 (3)	0.000 (2)	0.030 (3)	−0.003 (2)

### Geometric parameters (Å, °)

**Table:**

O1—C1	1.321 (6)	C4—C3	1.385 (5)
O1—H1	0.8200	C4—C5	1.384 (5)
O2—C1	1.207 (5)	C4—H4	0.9300
N1—C8	1.350 (5)	C7—C6	1.382 (6)
N1—C10	1.378 (5)	C7—H7	0.9300
N1—C5	1.423 (5)	C3—H3	0.9300
C9—C10	1.345 (6)	N2—C8	1.313 (5)
C9—N2	1.368 (6)	C6—C5	1.385 (5)
C9—H9	0.9300	C6—H6	0.9300
C2—C7	1.388 (6)	C8—H8	0.9300
C2—C3	1.388 (6)	C10—H10	0.9300
C2—C1	1.483 (6)		
			
C1—O1—H1	109.5	C2—C3—H3	119.6
C8—N1—C10	105.5 (4)	C8—N2—C9	105.1 (4)
C8—N1—C5	126.9 (3)	C7—C6—C5	119.5 (4)
C10—N1—C5	127.7 (3)	C7—C6—H6	120.3
C10—C9—N2	110.1 (4)	C5—C6—H6	120.3
C10—C9—H9	124.9	O2—C1—O1	123.1 (4)
N2—C9—H9	124.9	O2—C1—C2	122.8 (4)
C7—C2—C3	118.4 (4)	O1—C1—C2	114.1 (4)
C7—C2—C1	119.3 (4)	C4—C5—C6	120.0 (4)
C3—C2—C1	122.3 (4)	C4—C5—N1	119.5 (4)
C3—C4—C5	120.0 (4)	C6—C5—N1	120.5 (4)
C3—C4—H4	120.0	N2—C8—N1	112.5 (4)
C5—C4—H4	120.0	N2—C8—H8	123.7
C6—C7—C2	121.3 (4)	N1—C8—H8	123.7
C6—C7—H7	119.3	C9—C10—N1	106.8 (4)
C2—C7—H7	119.3	C9—C10—H10	126.6
C4—C3—C2	120.8 (4)	N1—C10—H10	126.6
C4—C3—H3	119.6		
			
C3—C2—C7—C6	−2.3 (7)	C7—C6—C5—C4	−0.1 (6)
C1—C2—C7—C6	178.7 (4)	C7—C6—C5—N1	−178.8 (4)
C5—C4—C3—C2	0.7 (6)	C8—N1—C5—C4	−14.9 (6)
C7—C2—C3—C4	1.0 (7)	C10—N1—C5—C4	167.0 (4)
C1—C2—C3—C4	179.9 (4)	C8—N1—C5—C6	163.9 (4)
C10—C9—N2—C8	0.1 (5)	C10—N1—C5—C6	−14.3 (7)
C2—C7—C6—C5	1.9 (7)	C9—N2—C8—N1	0.1 (5)
C7—C2—C1—O2	0.5 (7)	C10—N1—C8—N2	−0.3 (5)
C3—C2—C1—O2	−178.4 (5)	C5—N1—C8—N2	−178.7 (4)
C7—C2—C1—O1	−178.1 (5)	N2—C9—C10—N1	−0.3 (6)
C3—C2—C1—O1	3.0 (6)	C8—N1—C10—C9	0.4 (5)
C3—C4—C5—C6	−1.2 (6)	C5—N1—C10—C9	178.8 (4)
C3—C4—C5—N1	177.6 (4)		

### Hydrogen-bond geometry (Å, °)

**Table:**

*D*—H···*A*	*D*—H	H···*A*	*D*···*A*	*D*—H···*A*
O1—H1···N2^i^	0.82	1.83	2.645 (5)	178
C9—H9···O2^ii^	0.93	2.42	3.332 (6)	168

Symmetry codes: (i) *x*−1, −*y*, *z*+1/2; (ii) *x*+1, −*y*+1, *z*−1/2.
